# Hedgehog pathway inhibitors – current status and future prospects

**DOI:** 10.1186/1750-9378-7-29

**Published:** 2012-11-01

**Authors:** Asfandyar Sheikh, Arsalan Ahmad Alvi, Hafiz Muhammad Aslam, Abdul Haseeb

**Affiliations:** 1 Dow Medical College, Dow University of Health Sciences, Baba-e-Urdu Road, Karachi 74200, Pakistan

## Abstract

The Hedgehog (Hh) proteins comprise a group of secreted proteins that regulate cell growth, differentiation and survival. Inappropriate activation of the Hh signaling pathway has been implicated in the development of a variety of cancers. Hedgehog pathway inhibitors are a relatively new class of therapeutic agents that act by targeting the proteins involved in the regulation of Hh pathway (PTCH, SMO and Gli). Together, they serve as exciting new prospects, with a bright future, both alone or as an adjuvant to the more traditional anti-cancer drugs.

## Letter

The Hedgehog (Hh) proteins comprise a group of secreted proteins that regulate cell growth, differentiation and survival [1]. They are involved in organogenesis, and have been shown to promote adult stem cell proliferation [2,3]. Inappropriate activation of the Hh signaling pathway has been implicated in the development of several types of cancers including prostate, lung, pancreas, breast, brain and skin [4-9].

Sonic Hedgehog (Shh) is the best studied ligand of Hh pathway in vertebrates. In the absence of the ligand, the Patched (PTCH) receptor inhibits Smoothened (SMO), a downstream protein in the pathway. Binding of Shh to PTCH alleviates this inhibition, thus regulating the expression of Gli transcription factors [10]. Loss-of-function mutations of PTCH, gain-of-function mutations of SMO and misexpression of the Gli2 and Gli3 have been associated with tumor formation and maintenance in animal models of medulloblastoma and basal cell carcinoma of the skin [11-14]. Other studies have pointed towards Hedgehog signaling having an important role in angiogenesis (by increasing angiopoietin-1 and angiopoietin-2), metastasis (by increasing Snail expression) and suppression of apoptosis (by increasing Cyclins and anti-apoptotic factors and decreasing pro-apoptotic genes such as Fas) [15-18].

Hedgehog pathway inhibitors are a relatively new class of therapeutic agents that act by targeting the proteins involved in the regulation of Hh pathway. Cyclopamine is the prototype inhibitor of the Shh pathway that inactivates SMO by binding to its hepta-helical bundle [19]. It is currently undergoing preclinical and clinical studies as an anticancer agent in basal cell carcinoma, medulloblastoma and rhabdomyosarcoma [20,21]. Saridegib (IPI-926), a synthetic analog of cyclopamine, has shown positive results in Phase I clinical trial of advanced solid tumors [22]. Similarly, itraconazole, an antifungal drug, has also been shown to suppress growth of medulloblastoma in mice allograft models [23]. This compound acts as an SMO antagonist, in a manner distinct from its anti-lanosterol activity in fungi (other azole drugs have not been found to have this effect). Other candidates for future trials include Novartis’ LDE-225, Millennium Pharmaceuticals' TAK-441, Exelixis/Bristol-Myers Squibb's BMS-833923 (XL139) and Pfizer's PF-04449913 [24,25].

Vismodegib (IPI-926; Erivedge: Genentech, South St Francisco, CA, USA) has been recently approved by the FDA for treatment of advanced basal cell carcinoma [26]. However, like other drugs in the category, it also has an adverse effect profile. Due to its mechanism of action, it is contraindicated during pregnancy, as it is teratogenic, embryotoxic and fetotoxic [27]. Other adverse reactions include alopecia, muscle spasms, weight loss, fatigue, GIT disturbances and arthralgias [27].

The approval of Vismodegib by the FDA can prove to be the beginning of a new era in anti-cancer therapeutics. Other drugs targeting the Hh pathway are likely to follow. Together, they serve as exciting new prospects, with a bright future, both alone or as an adjuvant to the more traditional anti-cancer drugs.

## Competing interests

The authors declare that they have no conflict of interests.

## Authors’ contributions

AS was involved in choosing the topic and drafting the initial manuscript. HMA, AAA and AH were involved in critically revising the manuscript, listed in decreasing order of their contributions. The authors have read and approved the manuscript. The authors did not receive any financial support/grant.
